# A Clinically Oriented Introduction and Review on Finite Element Models of the Human Cochlea

**DOI:** 10.1155/2014/975070

**Published:** 2014-11-04

**Authors:** Dimitrios Kikidis, Athanasios Bibas

**Affiliations:** ^1^1st Department of Otorhinolaryngology, Head and Neck Surgery, National and Kapodistrian University of Athens, Hippocrateion General Hospital, 114 Vas. Sophias Avenue, 11527 Athens, Greece; ^2^UCL Ear Institute, 332 Grays Inn Road, London WC1X 8EE, UK

## Abstract

Due to the inaccessibility of the inner ear, direct in vivo information on cochlear mechanics is difficult to obtain. Mathematical modelling is a promising way to provide insight into the physiology and pathology of the cochlea. Finite element method (FEM) is one of the most popular discrete mathematical modelling techniques, mainly used in engineering that has been increasingly used to model the cochlea and its elements. The aim of this overview is to provide a brief introduction to the use of FEM in modelling and predicting the behavior of the cochlea in normal and pathological conditions. It will focus on methodological issues, modelling assumptions, simulation of clinical scenarios, and pathologies.

## 1. Background

Management of hearing loss requires a thorough understanding of the pathophysiological mechanisms through which diverse pathologies give rise to hearing impairment in humans. Furthermore when surgical interventions are required, planning and predicting of the surgical outcome require a thorough understanding of the ear mechanics. Despite significant progress in surgical techniques and auditory implant technologies, more work is needed to develop novel approaches to restore hearing that would take into account patient-specific anatomical and clinical data that could predict and maximise the final outcome.

Research into human inner ear physiology and pathology is usually clinical, with standard audiological and/or imaging studies. Noninvasive diagnostic tools are often unsatisfactory in either providing sufficient information on the physiology of hearing of individual patients or predicting the outcome of rehabilitation. Inability for in vivo measurements in the human cochlea greatly restricts the study of special aspects of cochlea physiology and pathophysiology. The only potential sources of raw data regarding cochlear mechanics in humans are temporal bone cadaveric experiments. Von Bekesy experiments on the travelling wave in human cadavers [1] are still highly influential, while more recently in vitro laser Doppler vibrometry (LDV) experiments in human temporal bones have provided more raw data on the vibrational characteristics of the basilar membrane (BM) as well as the spiral lamina [2].

Mathematical models of the cochlea can therefore play a key role in understanding the biomechanical processes involved in hearing. A series of such models have been developed during the past decades, towards a better understanding of ear physiology, including travelling wave production, outer hair cell motility, fluid dynamics, and micromechanics of the cochlea. The power of such a model is not only its ability to explain current physiological concepts and account for empirical (experimental and clinical) observations, but also its ability to incorporate future data, as they become available in the literature. By adjusting key parameters, cochlear models may also be used to simulate a variety of pathological conditions that lead to hearing loss as well as predict the cochlea's response when stimulated by auditory implants. It is safe to say that a complete computational model of the cochlea has yet to be attained.

The finite element method (FEM) is one of the most popular modelling techniques in structural mechanics. The basic concept is the subdivision of the domain of interest into components of simpler geometry called finite elements (discretization). These elements are interconnected at points common to two or more elements (nodes or nodal points) and/or boundary lines and/or surfaces. FEM encompasses all the methods for connecting many simple equations over those elements, to approximate a more complex equation over the domain of interest. The response of the mathematical model is then considered to be approximated by that of the discrete model obtained by connecting or assembling the collection of all elements. Apart from the governing equations of the system, boundary conditions should also be described in FE modelling, in which restrictions in the behaviour of the system (often in the form of a series of equations) at its physical border are considered. Although other modelling methodologies (i.e., the WKB-numeric method) have been successfully employed in modelling the cochlea, this paper will focus on FEM, as it is more intuitive to clinicians [3, 4].

## 2. Finite Element Modelling of the Normal Human Cochlea

### 2.1. Basilar Membrane

With a few exceptions, the majority of human cochlea modelling focuses on efforts to accurately simulate BM vibratory characteristics and travelling wave development, and thus this overview will largely concentrate on these modelling attempts. The usual approach is the use of 2-chamber (scala media included in scala vestibule), uncoiled, passive mechanical models. Thus, cochlear curvature, the micromechanics of the OC, and Reissner's and tectorial membrane are not accounted for [5–7]. Most of these models are also not coupled to the middle and outer ear and stimulation of the perilymph is simulated by applying the input pressure directly to the oval window. As the cochlea is universally modeled as a rigid structure, no fluid displacement occurs at its wall boundary and the normal component of fluid velocity at that point is zero. It is usually assumed that the pressure difference acts as a load on the BM causing it to deflect and the fluid velocity relative to the basilar membrane is zero at this interface. The perilymph is usually modeled as being incompressible and inviscid. However, inviscid flow may not be a valid assumption close to fluid boundary where the boundary layer plays a significant role, as in the space between the tectorial membrane and the reticular lamina.

A large number of parameters in regard to the physical characteristics and mechanical properties of the different structural elements are usually required for the construction of cochlear models (i.e., Young's modulus, biochemical and electrical properties, etc.). Not all of the relevant values are available in humans, so these either are borrowed from animal studies, are experimentally measured in vitro (usually by human temporal bone experiments), or have to be estimated through the comparison of model results with experimental studies. Overfitting may become a problem and thus reduce the predictive power of these models.

The BM is divided into two sections: (a) the arcuate zone, which consists of a single layer of radial fibers, and (b) the pectinate zone, which consists of a double layer of radial fibers in an amorphous ground substance. These fibers consist of collagen II without longitudinal cross links [8], so a highly orthotropic structure is assumed with high stiffness in the radial and low stiffness in the longitudinal direction. It is therefore important to include orthotropicity in BM modelling.

**Figure 1:**
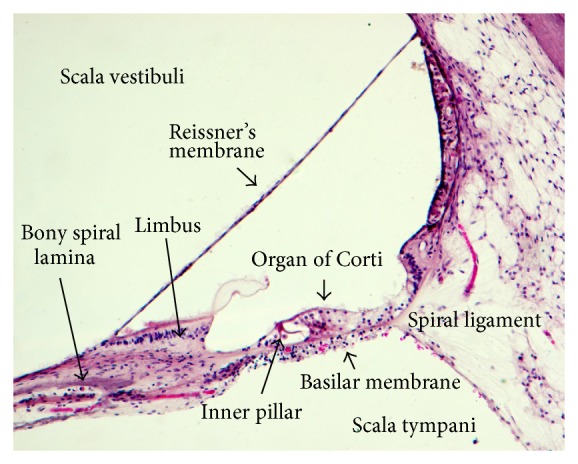
Human OC (approximately 12 mm from base). The osseous spiral lamina stops at the level of the insertion of the RM to the spiral limbus and is not in contact with the inner pillar. Thus, the OC rests entirely on the membranous spiral lamina (inner pilar) and the BM (specimen no. 280L, 10x, Human Temporal Bone Collection, UCL Ear Institute).

**Figure 2:**
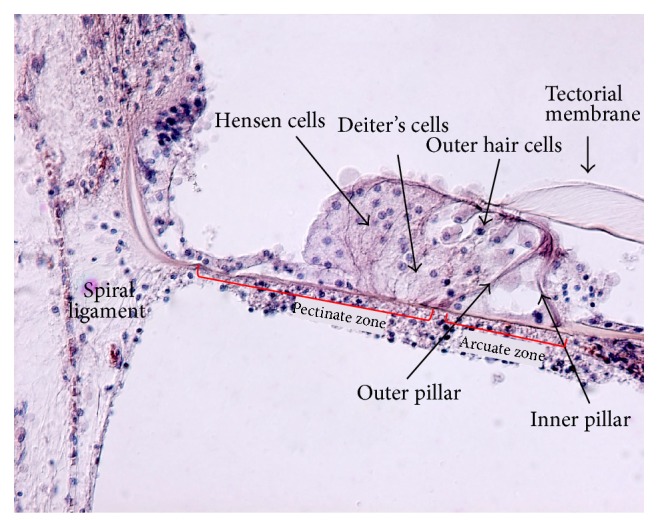
Human OC (approximately 12 mm from base). The fibers from the pectinate zone of the BM continue into the spiral ligament, where they are anchored (specimen no. F174R, 20x, Human Temporal Bone Collection, UCL Ear Institute).

The BM is usually modeled as attached to the spiral lamina and the lateral wall in a simply supported manner. However, the complex microanatomy of the medial insertion to the spiral lamina suggests that the spiral lamina support should not be modelled as a simple suspension. As is evident from histological sections (Figures 1 and 2), the main supporting bundles of the osseous spiral lamina continue directly into the BM fibers, suggesting that a clamped boundary condition may be more appropriate. It has also been shown that the osseous spiral lamina is not as rigid as previously thought and was found to vibrate similar to the BM, where the two are attached [2]. So far, cochlear models have not incorporated the flexibility of the osseous spiral lamina. Also, only few models take into account the fact that the lateral end of the BM is attached to the outer wall through the spiral ligament, which may also vibrate in response to sound. The fibers from the basilar membrane also continue directly into the spiral ligament, which again probably supports a clumped boundary condition rather than a simply supported one (Figure 2).

One of the first models to accurately simulate BM vibration characteristics was developed by Böhnke and Arnold [9]. It was a 2-chamber 3D finite element passive model constructed using microtomography. Notable boundary conditions were clamping of the spiral lamina inner boundary, allowing no rotation and displacement, and simple support at the side of the spiral ligament, allowing for rotation at the longitudinal (*x*) axis. Perilymph was considered inviscid. The BM dimensions were calculated using guinea pig values [10]. The input values were pressure loads of 1 Pa (equivalent to 94 dBSPL) at 3 frequencies (100 Hz, 2000 Hz, and 10000 Hz). The predictions of the BM displacement were in agreement with Von Bekesy's observations [1]. It was noted that the maximum displacement of the BM was of the same order of magnitude (0.2 nm) for the three frequencies tested.

Gan et al. [11] developed a passive uncoiled FE model of the cochlea, coupled to a model of the external canal and the middle ear. The same team developed later a more comprehensive model with a coiled cochlea [12]. Although the authors mention that information for the construction of the geometric model was obtained from temporal bone histology sections, it is apparent that actual 3D image reconstruction was not performed from the available sections, at least regarding the cochlea. The authors were able to compute the middle ear transfer function and the BM vibration displacement normalised by the stapes displacement as a function of the distance from base. Although validation for the middle ear transfer function was achieved by experimental data from human cadaver ears, it is not clear which data were used for the validation of the BM response. The model was also used to compute the efficiency of the forward and reverse mechanical driving with middle ear implant, as well as the passive vibration of the BM when a cochlear implant was placed in the scala tympani. This is probably the only FE model where RM is taken into account. The introduction of the scala media in their model allowed for a more realistic representation of the physical properties of the cochlea. Scala media modelling introduced an asymmetry between the forward and reverse driving of the cochlea. Thus, it was also used to predict how its mechanical properties affected the vibratory characteristics of the BM. According to the authors, the model may be used to predict how changes in the morphology or function (permeability) of the RM can affect cochlear function.

### 2.2. Organ of Corti

The OC was initially modeled as a rigid structure, but FE methods were employed later to capture its micromechanical properties in more detail [9, 13, 14]. However, these models are either 2D or 3D but of a limited length along the* x*-axis, which limits their ability to accurately predict the coupled response of the basilar membrane.

In most models, the osseous spiral lamina is seen to extend to the inner pillar cells, as observed in animals. This is in contrast to the mobile outer pillar cell that rests on the BM. In this way, the base of the inner pillar cell would have a hinge-like action at the point of contact with the bony spiral lamina. However, this relationship does not exist in humans, where the bony spiral lamina has no such spread, and it falls short of reaching the attachment of the RM to the spiral limbus (Figure 1). Thus, in the human cochlea, the entire OC rests on the membranous spiral lamina or the BM.

Böhnke and Arnold [9] designed one of the first 3D models of the OC to account for the active function of the OHCs. The ability of the OHCs to elongate and contract was modeled by heating and cooling according to a thermal expansion coefficient *a* = 10^−4^. This feature could be switched on or off to simulate the active or passive OC, respectively. To achieve an effective gain of 40 dB for the displacement of the reticular lamina, OHCs gain of 2 (i.e., the ratio of somatic OHCs length change to stereocilia bundle displacement) was chosen to reflect experimental results [15]. When the active feature of the OHCs was considered, an additional increase of the reticular displacement (40 dB, amplitude = 160 nm) with additional distortion of the BM was observed.

Nam and Fettiplace [16] presented a three-dimensional model to illustrate deformation of the OC by the two active processes: voltage-driven contractility of the outer hair cell body and active motion of the hair bundle. The model was able to capture isolated local responses with simulated longitudinal lengths of 400 and 600 *μ*m at the base and apex. In some simulations, lengths of 1200 mm were used. This is probably the only model incorporating such a long length in their OC model, providing a limited coupled response between the OHCs and the BM. The radial structural components were coupled in the longitudinal direction by three continuous longitudinal structures and one discrete longitudinal structure. Continuous longitudinal coupling included longitudinal beams of BM, reticular lamina, and tectorial membrane, whereas the coupling by the OHCs and Deiter's cell phalangeal processes was discrete. Outer hair cells and Deiter's cells were tilted in opposite directions, and this is one of the few models that takes into account the phalangeal attachments to accurately reflect their microanatomic orientation [17]. The radial stiffness of the tectorial membrane was found to have an important effect in mechanical feedback.

### 2.3. Outer Hair Cell Modelling

The mechanisms underlying cochlear amplification include the prestin-mediated somatic motility of the OHCs as well as active movements of the hair bundles contributed by the mechanotransducer channels (stereociliary motility) [18]. Spector et al. [19] modeled the OHCs taking into account the angle of their inclination as well as their length, so that they could simulate the active process in the cochlea, whereby two cross sections along the cochlea are coupled through the connection of the same column of active OHCs. One of the advantages of this approach was the use of coefficients to represent these active forces. Pathological conditions that may affect the mechanoelectrical transduction (i.e., presbyacusis) can thus be modelled by this approach, by modifying these coefficients.

Stereocilia hair bundles are not usually meshed but are included as single elements. Duncan and Grant [20] developed a finite element model taking into account the stiffness and deflection properties of the hair bundle, but without coupling to the rest of the OC. The main boundary condition was the anchorage of each cilium at its base. Loading of the model was simulated by a single static force applied to the tip of the tallest cilium. Tips and lateral links were removed in turn from the model to assess their effect on bundle stiffness. When the tip links were removed, the overall bundle stiffness reduced by 55%.

As mentioned previously, the cochlea is usually uncoiled in most modelling approaches, as coiling was reported to have a negligible effect on cochlear micromechanics. However, recent modelling suggests that, by taking into account the curvature of the cochlear duct, the shear gain of the cochlear partition at the apex is increased, which is an indirect measure of the bending magnitude of the OHCs stereocilia [16]. This effect is not observed in the basal region of the cochlea, and it seems that it is the radius of curvature that is the main parameter responsible for the difference in this effect. One of the limitations of this study was that calculations were based only on the relative motions of the cochlear structures and not on the absolute amplitude of the BM vibration.

### 2.4. Tectorial Membrane

Gueta et al. [21] investigated the effect of the anisotropic properties of the tectorial membrane on the deflection of the stereocilia. By first using force spectroscopy, it was found that the stiffness of the tectorial membrane was significantly larger in the vertical than the lateral axis. Consequently, it is more resistant to the vertical motion of stereocilia and is therefore deflected laterally when pushed against. This was confirmed by FE simulations of the interaction between the stereocilia and the tectorial membrane, when this anisotropy was incorporated into the model, thus providing an additional mechanism for the deflection of outer hair cell stereocilia apart from viscous fluid forces. It is suggested by the authors that the coexistence of both mechanisms may enhance the lateral deflection of the OHCs stereocilia.

Gavara and Chadwick [22] explored the difference in anisotropy between the radial and longitudinal axis of the tectorial membrane based on the anisotropy caused by the orientation of the collagen fibres. It was found that tectorial membrane deformations are more pronounced in the direction parallel to the collagen fibers (radial direction) than perpendicular to them (longitudinal direction). It is therefore assumed that there is a strong coupling of the three OHCs in the same radial direction, while OHCs placed at a different longitudinal direction remain uncoupled. However, other models stress the importance of longitudinal coupling of the tectorial membrane, which allows for interaction between cells placed at different places along the BM, and are able to closely replicate the amplitude of BM traveling wave in vivo [23, 24].

### 2.5. Reissner's Membrane

As described above, only Zhang and Gan [12] have included the RM and the scala media in their model and the way they affect BM vibratory characteristics. Apart from its effect on the BM, Reichenbach et al. [25] have recently described travelling waves on RM. By using scanning laser Doppler vibrometry on the RM of a variety of animal species, travelling waves were measured, thus supporting a theory on its role in otoacoustic emissions.

### 2.6. Round Window

Modelling the round window membrane is of particular importance as middle ear implants have been used for directly exciting the cochlea in cases of mixed and severe conductive hearing loss. It has been recently shown that mechanical stimulation of the round window membrane with an active middle ear prosthesis produces cochlear microphonics and stapes velocities that are functionally equivalent to acoustic stimulation [26]. Zhang and Gan [12] examined the mechanical properties of the human round window membrane using both acoustic stimulation and laser Doppler vibrometry measurements in human temporal bones. A FEM was subsequently used to determine the complex and relaxation moduli, by using an inverse problem solving method. It was shown that the average storage modulus changes from 2.32 to 3.83 MPa and the average loss modulus from 0.085 to 0.925 MPa for frequencies between 200 and 8000 Hz.

### 2.7. Input to the Cochlea and Middle Ear Coupling

The physiological motion of the stapes footplate is piston-like at low frequencies but shifts to a rocking movement around its short and long axes at higher frequencies. However, most current human cochlear models do not consider the effects of this rocking motion because it is believed not to contribute to considerable fluid volume displacement so as to have an effect on cochlear mechanics.

Models developed by Kim et al. [27, 28] and Zhang and Gan [12] have coupled FE models of the external and middle ears to the cochlea. Modelling the stapedial annular ligament is important as the oval window is input site for the excitation signal. Gan et al. [29] constructed an FE model of the stapedial annular ligament that they used together with experimental setup in temporal bones to calculate its mechanical properties. Using a micromechanical testing system, the shear modulus of the annular ligament was calculated to change from 3.6 to 220 kPa when the shear stress increases from 2 to 140 kPa. A 3D finite element model of the experimental setup with the stapedial annular ligament was created for assessing the effects of loading variation and measurement errors on results. A fixed boundary condition was applied at the periphery of the annular ligament to simulate its attachment to the oval window, and the stimulating force was applied at the center of stapes head perpendicular to the stapes footplate. The FE modeling results confirmed that simple shear dominated the deformation of the annular ligament.

### 2.8. Validation Methodology of Existing Models

Clinical validation of cochlear models is not straightforward, since there are no direct noninvasive techniques to measure the BM responses of the human cochlea in vivo. To the best of our knowledge, no cochlear models have used true clinical validation. The most commonly used validation approach in cochlear modelling is the comparison with Von Bekesy's experimental data BM displacement and the distribution of the characteristic frequency as described by Greenwood [30]. Experimentally determined amplitude-frequency curves by Stenfelt et al. [2] and Gundersen et al. [31] have also been used. However, all these measurements involve the passive cochlea, so the active cochlear processes cannot be validated. BM velocity and amplitude measurements from different species have also been used for validation using laser Doppler vibrometry.

Novel validation approaches could include the use of cochlear impedance and psychophysical masking data, which, to the best of our knowledge, have not been currently described in the literature. Cochlear impedance and intracochlear sound pressure measurement data provide a means to validate cochlear models acoustically as a whole system. To the best of our knowledge, no FEM model has yet been verified against these important acoustical properties. For a quantitative model validation, intracochlear pressure data exist from human cadaver cochleae. Nakajima et al. performed simultaneous sound pressure measurements in scala vestibuli and scala tympani of the cochlea in human cadaveric temporal bones and measured the transfer functions of scala vestibuli and scala tympani pressures relative to the ear canal pressure [32]. The pressure in scala vestibuli was generally 10–20 dB larger than in scala tympani over a wide range of frequencies. For frequencies below 500 Hz, the phase of the scala tympani pressure relative to ear canal pressure was slightly less than zero, while the phase of the scala vestibuli pressure had almost a *π*/4 lead. Above 500 Hz, the phases of the pressures were similar. Nakajima et al. were also able to measure the differential impedance across the cochlear impedance. This has the advantage that, in contrast to standard cochlear impedance measurements, the round window impedance is not taken into account, and hence these data can be used for validation in models where the cochlea is not coupled to the middle ear.

Psychoacoustic masking methods are widely used to estimate some response properties of the human BM [33–36]. The resulting psychoacoustic tuning curves (PTCs) are thought of as isoresponse curves and are assumed to correspond to BM tuning (or isoresponse) curves. Consequently, it is assumed that the tip frequency of any given PTC matches approximately that of a corresponding BM tuning curve and thus could be used for the validation of the human cochlear models.

## 3. Finite Element Modelling in Clinical Scenarios and Applications

Apart from simulating normal physiology, one of the most powerful uses of models is their ability to predict pathological conditions and to simulate surgical management. What follows is a brief review of some of the papers that have used FE cochlear models to simulate clinical pathologies and surgical applications.

### 3.1. Basilar Membrane Pathology

Skrodzka [37] used a cochlear FEM to predict travelling wave patterns and characteristic frequencies by simulating BM structural damage. Mechanical damage to the BM (rupture) may result following acoustic trauma (leading to permanent sensorineural hearing loss) or cochlear implant insertion (leading to decreased performance in cases of combined electrical-acoustic stimulation strategies). The BM rupture was modeled as a hole centered at a point 7 mm from the basal end. An additional mass was added in the same place, increasing the BM thickness by a factor of 10. The main excitation frequency used in modified BM models testing was 5 kHz. The model predicted that the structural alterations of the BM affected velocity values and location of the maximum displacement. Interestingly, some BM locations were predicted to resonate in more than one frequency. These predictions have not been validated clinically.

### 3.2. Semicircular Canal Dehiscence

Kim et al. [38] modeled superior semicircular canal dehiscences (SSCD) in a passive coiled 3D FE model, by removing a section of the outer bony wall of the canal. The geometric reconstruction was obtained by using mCT and the cochlea was coupled to the middle ear. The following modelling assumptions were taken into account: (1) perilymph was considered inviscid, (2) the BM had orthotropic elasticity, and (3) damping was accounted for by a complex Young's modulus. A zero-pressure boundary condition was applied to the area exposed by the dehiscence, since at this point the perilymph is in contact with the cerebrospinal fluid, which is much larger in volume. BM velocities were calculated in different frequencies, for both AC and BC stimulation, and hearing loss was calculated as the difference in the maximum amplitude of BM velocity between the pre- and postdehiscence simulations. BC stimulation was simulated by applying rigid-body vibrations along arbitrary directions in the three-dimensional plane. The model predictions were consistent with the audiometric thresholds of patients with a SSCD, showing an improvement in bone conduction and elevation of air conduction thresholds at frequencies below 1 kHz, although the variation in clinical presentation is high. Additional information derived from the model was the importance of the width of the dehiscence closest to the oval window.

### 3.3. Stapedotomy

Kwacz et al. [39] used an FE model of the uncoiled cochlea to study the influence of stapedotomy on round window membrane vibration and to estimate the postoperative outcome. The model was validated against experimental measurements from cadaver temporal bones using laser Doppler vibrometry. The reduction in the RW membrane vibration amplitude was related to the piston diameter, and the maximum reduction was achieved at the highest frequencies (>3 kHz). This reduction corresponds to an incomplete closure of the air-bone gap resulting in residual conductive hearing loss after stapedotomy. The authors also used the FE model to test a new prosthesis that they developed in their laboratory.

### 3.4. Middle Ear and Cochlear Implantation

Finite element models of the cochlea are very useful to surgeons for enhancing their understanding of the mechanics and stresses involved when placing different auditory implants. Simulations are also important for manufactory designers to select appropriate mechanical profiles for future implants.

Zhang and Gan [12] simulated the efficiency of middle ear transducer (MET) on different coupling conditions. It was thus shown that coupling of the MET to the round window membrane (reverse driving) was more efficient than coupling to the ossicles (forward driving), as far as BM vibration amplitude was concerned. This was attributed to the smaller distance to the BM, through the RW stimulation.

FE simulations of cochlea implantation have been used to predict BM vibration patterns after electrode insertion as well as predict trauma to the cochlea by using different electrode mechanical properties. Zhang and Gan [12] showed that cochlear implant insertion resulted in a change in the frequency response of the BM, by shifting the peak response towards the apex. Passive vibration of the BM above the implant was also greatly diminished. The BM vibration was eliminated at high frequencies and remained at certain level at low frequencies (below 1 KHz). The results show that the passive vibration of BM was preserved at low frequency. Chen et al. [40] used a FE model of the cochlea to predict the insertion trajectory and contact pressures of a straight cochlear implant electrode array from nucleus. It was predicted that an electrode with a uniform stiffness would produce higher contact pressures at the tip (and hence significant more damage at insertion) than a graded stiffness electrode. Kha and Chen [41] used a FE model to predict the damage to the BM caused by the proximal end of the cochlear implant array. It was predicted that the contour array design is most likely to impinge on the BM, compared with the straight array and the single wire electrode, by exerting higher stresses on the scala tympani's upper surface. In another study by Lim et al. [42], a 3D FE model of the cochlea was used to evaluate six electrodes with different stiffness properties due to different wire arrangements. It was found that the contact pressure at the tip and the insertion force were highest when the wires were arranged horizontally. The cochlear implant insertion technique was simulated in a FE analysis of the cochlea by Kha et al. [43]. It was predicted that anticlockwise rotations between 251° and 901° applied at the basal end of the array (on the right side) significantly reduce stress trauma on the BM which support the practice of applying small rotation partway through insertion of electrode array to minimize damage to the BM.

## 4. Identification of Beyond the State of the Art Characteristics for Future Modelling

### 4.1. Multiscale Modelling

Multiscale modelling involves solving physical or mechanical problems, which have distinct features at multiple (spatial or temporal) scales. The main aim of multiscale modelling is the calculation or computation of mechanical properties or system behavior at one level using information or models from higher or lower levels. Although combining models at different scales may help to model complex structures, the combination of models will only be as accurate as the least accurate model in the combination. Multilevel modelling has already been employed in other physiological systems, mainly in the cardiovascular domain [44–49]. Multiscale modelling can also extend and include pathological processes. To the best of our knowledge, there is currently no multiscale FE model of the entire cochlea. Steele et al. [50] have published a multiscale model of the OC. However the article focuses on the multiscale differences in the mechanical properties (mainly stiffness related) at scales in the OC (vibrating cochlear partition, the hair cells, and stereocilia).

### 4.2. Patient-Specific Modelling

Patient-specific modelling may be proved useful in predicting outcome following medical or surgical intervention and it has been employed in various medical and biological domains. In orthopedics, for example, it may provide surgeons with data regarding bone stress distribution or implant microdisplacements [51]. It may also be used to provide enhanced information from imaging data regarding risk fracture prediction. In interventional cardiology, it has been recently used to investigate the use of FE analysis on the impact of carotid stent placement by using data from individual patients [52]. To date, there are no patient-specific models of the cochlea or the ear.

## 5. Conclusions

Finite element method is a powerful modelling approach for structural mechanics, and as such it has been proved useful in the study of cochlear mechanics, in both health and disease. As far as clinical applications are concerned, there is a good agreement between model predictions and clinical data, especially regarding superior semicircular canal dehiscence, stapedotomy, and auditory implantation. One of the main restrictions of the current FEM models is the arbitrary or approximate use of parameter values, as well as the difficulty in proper clinical validation. Although model parameter values can be fine-tuned so that model behavior matches clinical data, it may not be possible to demonstrate that the models are physiologically realistic. On the other hand, in vivo measurement of basilar membrane vibration is currently not feasible, and hence validation methodology is restricted to in vitro measurements in temporal bones. Future attempts should focus on multiscale and patient-specific modelling as well as the ability to couple cochlear models with models of the central auditory pathway.
